# A bibliometric analysis of micro electro mechanical system energy harvester research

**DOI:** 10.1016/j.heliyon.2021.e06406

**Published:** 2021-03-08

**Authors:** Ida Hamidah, Roer Eka Pawinanto, Budi Mulyanti, Jumril Yunas

**Affiliations:** a Department of Mechanical Engineering Education, Universitas Pendidikan Indonesia, Jl. Dr. Setiabudhi 207, Bandung, 40154, Indonesia; b Department of Electrical Engineering Education, Universitas Pendidikan Indonesia, Jl. Dr. Setiabudhi 207, Bandung, 40154, Indonesia; c Institute of Microengineering and Microelectronics, Universiti Kebangsaan Malaysia, Bangi, 43600, Selangor, Malaysia

**Keywords:** Bibliometric, MEMS, Energy harvester, VOSviewer

## Abstract

Micro Electro Mechanical System (MEMS) energy harvester's research interests have been increasing rapidly, indicating that the topic has given significant contributions to the sustainable development of energy alternatives. Although many research activities have been conducted and reported since several years ago, only limited efforts have been made to analyze the research's impact in this area. In this paper, we report a bibliometric analysis on the research progress in MEMS for energy harvester. VOSviewer software is used to support the analyst that includes the distributions of the publication journals, authors, affiliations and the highly cited papers reporting the progress as well as the frequency of keywords and their relationships found in the search engine. The analysis is mainly aimed to identify the research map based on publication reports. 1772 papers were initially identified and summarized based on the analysis on three focused mainstream research topics in MEMS for alternative energy, such as MEMS energy harvester, power harvesting and energy scavenging, other term analogies to MEMS such as micromachines and microsystem were included in the analysis parameter. As a result, it is found that the study on the MEMS energy harvester is mostly categorized in the engineering field, while China has been conducting the most projects. The Journal MEMS and Journal of Micromechanics and Microengineering have been the most journals publishing reports on MEMS energy harvester's research progress. Based on these analyses, some potential issues in future MEMS energy harvester research have been identified, including the contributions of new materials, the MEMS new structure's involvement, and the optimization of the vibration concepts and principles of MEMS energy harvester. These analyses would give an overview on the progress of the development and improvement in MEMS energy harvester and give a proper guideline for future MEMS research in the energy field.

## 1 Introduction

In the last decade, the research interests in Micro Electro Mechanical System (MEMS) devices in energy harvester have been increasing rapidly and became one of the most exciting trends of the future MEMS sensor application (Zhu et al., 2020). The topic of MEMS for energy harvester has emerged to remarkably a new topic regarding the application and its demand to provide alternative energy sources for the electrical power delivery of various microdevices (Sun et al., 2018; Liu et al., 2018; Yunas et al., 2020a). This trend resulted in a tremendous increasing number of publications that are very useful for further developing the device.

Several research activities have been conducted and reported since several years ago aimed to expose the progress of the development and improvement in MEMS energy harvester, especially for biomedical, automotive and consumer electronics application (Hannan et al., 2014; Naito and Uenishi, 2019; Gljuscic et al., 2019).

Some other studies had reported the research progress on MEMS energy harvesting utilizing piezoelectric (Shi et al., 2016); electrostatic (Oxaal et al., 2016) and multi-frequency mechanical vibration-based electromagnetic energy conversion (Liu et al., 2013). Meanwhile, the investigations on the functional material, such as polymer composites applied to an electromagnetic actuator (Yunas et al., 2020b) and the expansion of the MEMS energy harvester into the internet of things system (IoT) (Duque et al., 2019; Iannacci, 2019) have enriched the literature significantly in the field of MEMS energy harvester.

However, limited efforts have been made to analyze the impact of the research in this area. It also became hard to understand the research progress on MEMS energy harvester topic, as the publications over the last decade have been significantly increased, even though there is no correct guideline of the directions. Therefore, to obtain proper guidelines for the future development and research direction of MEMS energy harvester, it is crucial to systematically inspect MEMS energy harvester's research progress through literature analysis. One of the effective methodologies to understand the research roadmap is the review of the publication that highlights the current research and development in a field of concern and provides some direction for future study. It also can guide the other scholar to avoid repetition or research duplication (Rahman et al., 2020; Zhang and Yuan, 2019; Zhao et al., 2019).

Despite some critical reviews on MEMS have given clear overviews on the progress of energy harvester research (Bathrinarayanan and Tamizhchelvan, 2014; Hu and Liu., 2015; Avila-Robinson and Miyazaki., 2013), bibliometric analytics literature is still powerful and useful for a more systematic approach resulting in-depth analysis on the research progresses (Gopalakrishnan et al., 2015; Elango et al., 2013). Bibliometric is a research method for conducting quantitative analysis of a document in a specific research field (Du et al., 2014; Ran, 2005; Mao et al., 2015). The analysis refers to evaluating bibliographic scientific research publications data following quantitative and qualitative statistical methods. The analysis including bibliographic mapping, profiling of publications, clustering, and visualizing the published works (De Bakker et al., 2005). Moreover, this analysis proffers a useful indication to the expert for exploring dominant interrelationship in the specific literature (Fellnhofer, 2019) and the impact of topics, journals, researchers, countries, and institutions (Krauskopf, 2018). It can help researchers predict future research trends (Zhou et al., 2007).

Bibliometric applies statistical and mathematical methods to investigate several characteristics of documents such as co-relationship, distributed architecture, and diverging schemes (Dong et al., 2014). The Bibliometric analysis can also evaluate various aspects of a unique research topic (Keiser and Utzinger, 2005) Bibliometric have been used to analyze the climate change vulnerability (Wang et al., 2014), research on haze (Li et al., 2017), research on Covid-19 (Hamidah et al., 2020), and research on low carbon education (Hudha et al., 2020).

No study analyzes the MEMS energy harvester research activity based on bibliometric metrics information to the prime of our knowledge. Therefore, it is crucial to explore MEMS energy harvester's research by identifying and summarizing MEMS energy harvester's entire contexts from the last ten years. The current study of MEMS energy harvester literature using bibliometric analytical techniques will help evaluate MEMS energy harvester research's impact. Furthermore, based on the published paper's analysis about MEMS energy harvester papers, the potential directions and trends are predicted.

## 2 Research methodology

### 2.1 Literature screening

A holistic study summarizes the research output from 2009 to 2020 in MEMS energy harvester published in Scopus. The entire paper screening process comprises three steps. Firstly, all articles about MEMS energy published in Scopus is collected as initial input, which is based on the title, abstract and keywords of any terms related to MEMS (such as microelectromechanical systems or micromachines or microsystem technology) and any terms related to energy harvester (such as power harvesting or energy scavenging). The time frame of the publication is limited from 2009 to 2020. Secondly, 1772 papers were initially identified covering various fields such as physics and astronomy, engineering, computer science, material science, mathematics, and energy. Thirdly, several categories, such as journal articles, conference papers, books, reviews, and other publication types for discussion and further analysis, are distinguished by the initially identified papers.

### 2.2 The analysis tool: VOSviewer software

VOSviewer software is commonly used to visualize and analyze a bibliometric network developed by Van and Waltman (2010). The VOSviewer had been used in analyzing the bibliographic map on the energy performance (Zhang and Yuan, 2019), reviewing the bibliographic data of building control (Park and Nagy, 2018), and analyzing citation-based clustering in the field of astronomy and astrophysics (Van and Waltman, 2017). This study uses this software to make several visualization maps, including keyword co-occurrence, co-authorship, citation, and co-citation map based on bibliographic data.

Every circle in the VOSviewer visual map shows a term. The circle and text size show the term activity. The large circle and text indicate the terms that are preferred in a field. The distance between the two terms indicates the level of relationship between them. For this instance, if the distance between two terms is small, the relationship between them more robust.

A systematic mapping study (SMS) approach proposed by Fellnhofer (2019) had been adopted in this paper. Bibliometric coupling and direct citation analyses are considered the most credible and precise technique in mapping the study literature. Also, SMS is capable of clustering and visualizing interconnected contributions and contributors' networks.

The articles from the Scopus database had been downloaded then analyzed using VOSviewer and MS Excel. Remarkably, the bibliography information had been overviewed then analyzed using MS Excel. The clusters of the data were also formed by VOSviewer software (Paltrinieri et al., 2019). The clusters' theme is identified by evaluating each publication's titles, abstracts, and keywords within the cluster. Table 1 highlights the analysis step in this study.

**Table 1:** Step of bibliometric analysis.

Steps	Database/Programs	Remarks
1. Data collection	Scopus database	Timespan: 2009 to 2020 with keywords/terms, title, abstract (“microelectromechanical systems or MEMS or micro-electro-mechanical systems or micromachines or microsystem technology and energy harvester or power harvesting or energy scavenging”). 1772 documents related to the MEMS energy harvester have appeared. Type of document: Journal article. 672 articles appeared for further analysis.
2. Analysis and identification	VOSviewer	672 documents were automatically identified for bibliometric coupling analysis.
3. Further analysis and map	MS Excel, VOSviewer	Descriptive analysis of bibliometric information; Co-authorship of affiliations and country, Co-occurrence keywords, Co-citation of cited references, and journals
4. Research topic interpretation	Qualitative interpretation	The authors reviewed the titles, abstracts, and keywords and analyzed of each research topic.

## 3 Results, analysis and discussion

### 3.1 Classification by main research fields

Based on the collected papers' classifications, 672 papers appeared when MEMS energy harvester, micromachine energy harvester, and microsystem energy harvester were typed distributed in various fields engineering, physics, and astronomy, material science, computer science, and energy. In this phase, the paper classification screening is carried out by title, abstract, and keywords.

**Figure 1:**
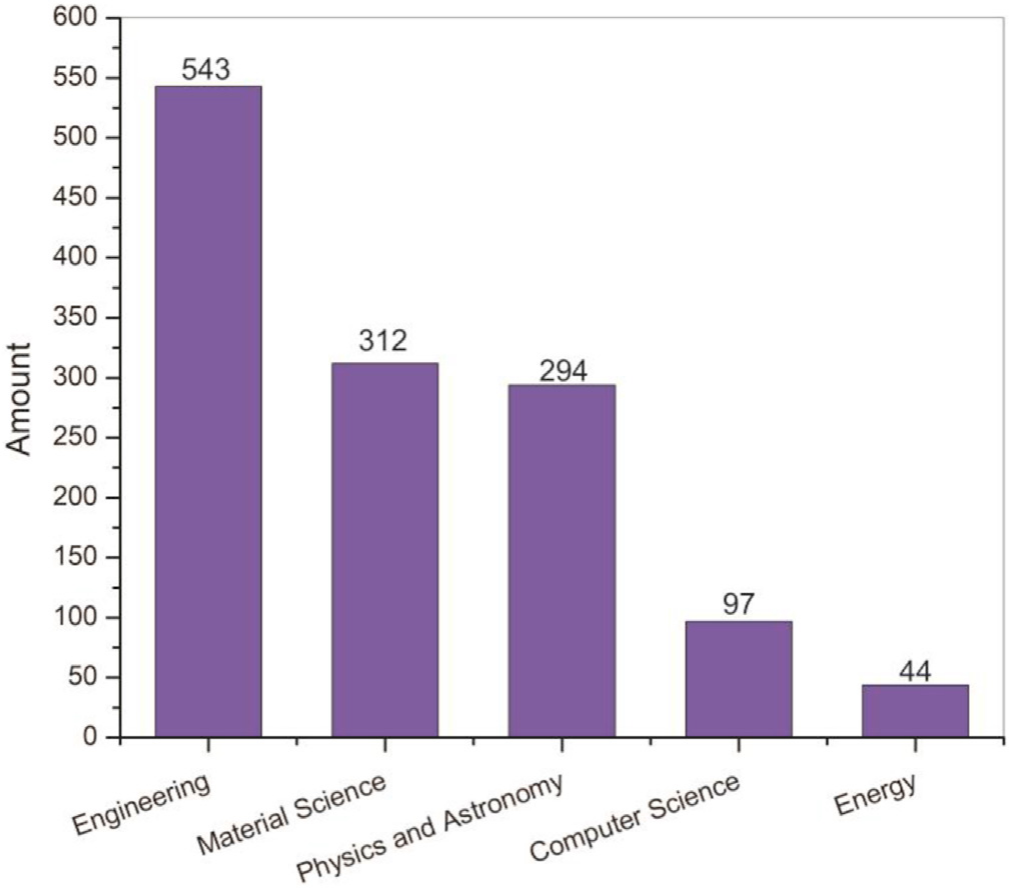
Top 5 research fields in MEMS energy harvester.

Nevertheless, it is difficult to investigate a paper in a particular field, because a paper contains interdisciplinary issues in the MEMS energy harvesting topic. For example, the main topic is related to engineering, but physics and materials science is also important to be analyzed (Lu et al., 2018a, 2018b). Thus, the paper is classified into engineering, material science, and physics fields. According to this rule, the top 5 fields in MEMS energy harvester research from 2009 to 2020 were identified in Figure 1. It is shown that the research was covering various areas such as engineering, material science, physics and astronomy, computer science, and energy. Therefore, it is shown that the three areas of most publications including engineering with 543 papers (80.8% of the total screened papers) followed by material science and physics and astronomy field.

It is also clear that materials and physics show more publications than those of energy, which shows that basic science still dominates this research activity. Meanwhile, it is worthy to note that MEMS energy harvester research is remarkably interdisciplinary, which is shown by many papers is related to more than one area.

### 3.2 Countries researching in MEMS energy harvester

**Figure 2:**
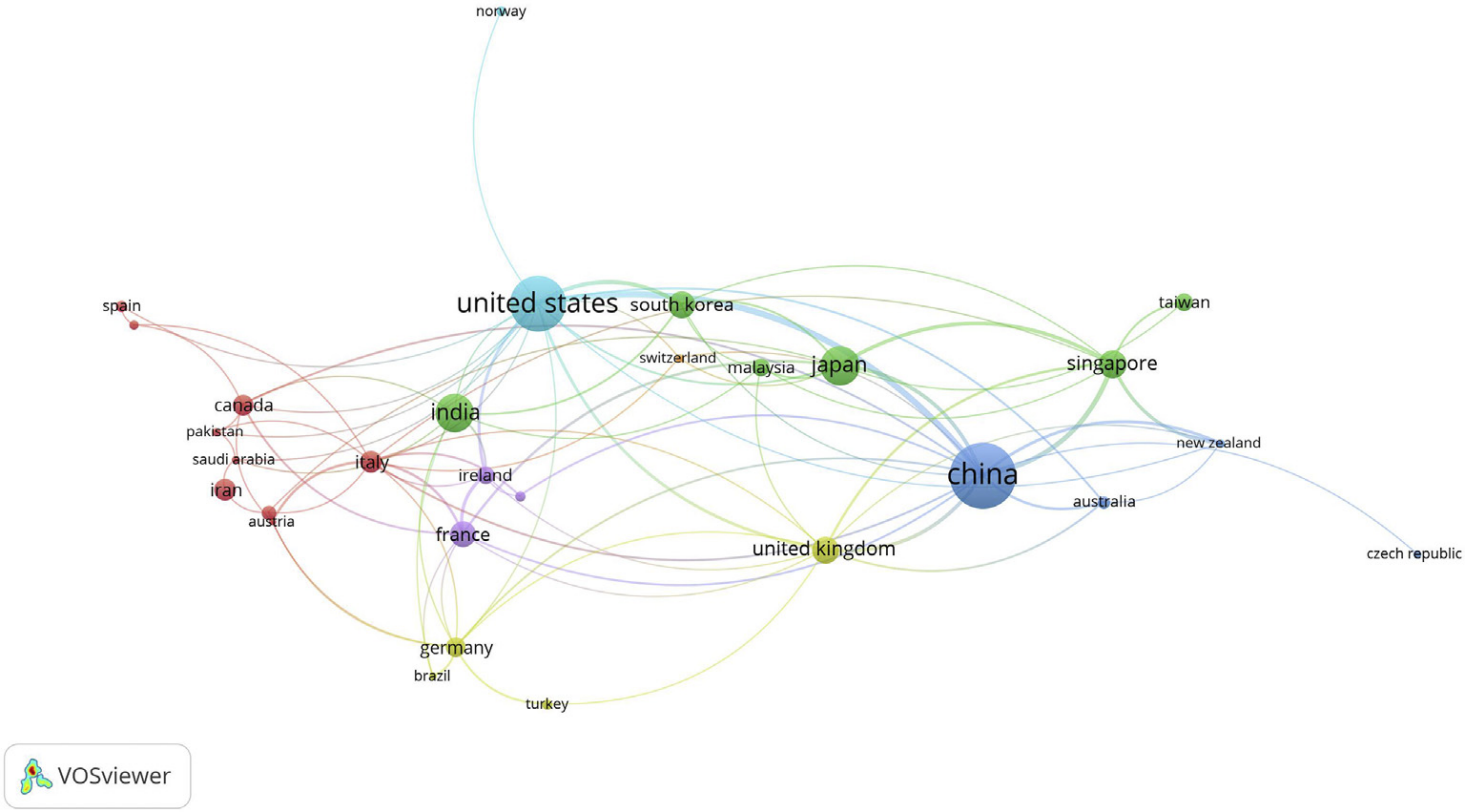
The visualization of a network between countries in MEMS energy harvester research.

Since the MEMS component of electronic devices become smaller and compact, the energy source to operate the MEMS device also becomes a global concern. Therefore, it is essential to analyze the papers published in various countries related to energy harvesting device research. Figure 2 shows the distribution of countries where the research in MEMS energy harvester is conducted. China is the country most active in conducting MEMS energy harvesting studies over the past decade. Chinese researchers contributed 145 papers, or 21.6% of the total papers analyzed. It is reasonable because the demand for MEMS devices in China has grown fast in the last decade; thus, it is directly related to MEMS energy harvester needs (Chunbo, 2018; Lien and Shen., 2014). Meanwhile, US researchers contributed 112 papers with a percentage of 16.7%. Moreover, the European region contributed 205 papers over the last decade. The synergy between countries across Europe in MEMS energy harvester is based on lines-connection, as shown in Figure 2.

### 3.3 Main journal publishing MEMS energy harvester research

**Figure 3:**
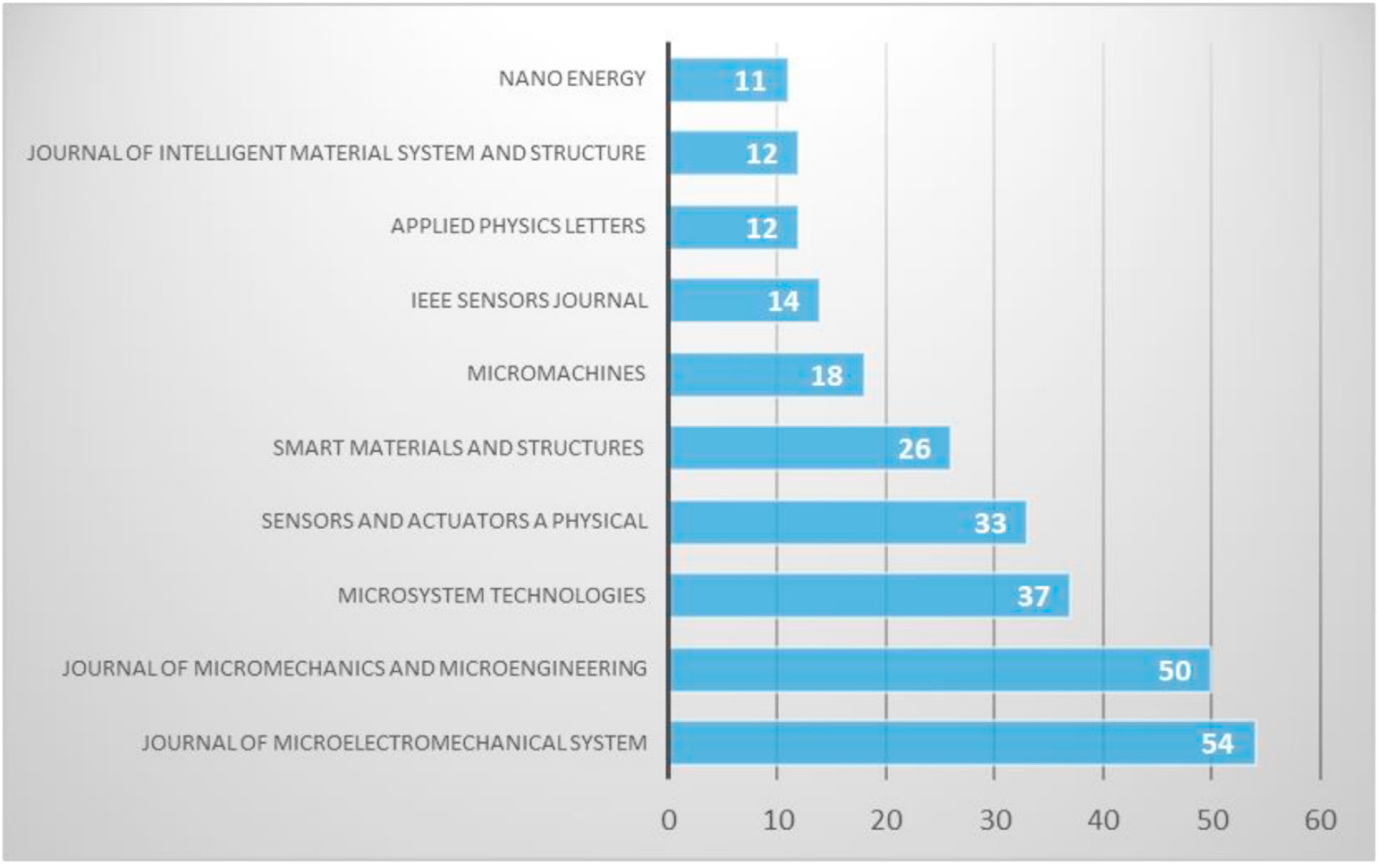
Top 10 journals publishing of MEMS energy harvester research.

As previously noted, the MEMS energy harvester topic can be linked to interdisciplinary fields and papers published in several journals. The journal publishing MEMS energy harvester was analyzed based on the number of publications in specific journals. Figure 3 shows that the top 10 most published journals in MEMS energy harvester research are the Journal of Microelectromechanical System and Journal of Micromechanics and Microengineering, with a total of 54 and 50 articles, respectively. Meanwhile, the other journals which contributing to MEMS energy harvester research include Microsystem Technologies (37 papers), Sensors and Actuators: A Physical (33 papers), Smart Materials and Structures (26 papers), Micromachines (18 papers), IEEE Sensors Journal (14 papers), Applied Physics Letters (12 papers), Journal of Intelligent Material System and Structure (12) and Nano Energy (11 papers).

### 3.4 Analysis of main author contributions

A review of the scholars authoring the 672 MEMS energy harvester papers was used to identify the most productive authors, including their co-author networks' connections. For this analysis, five papers are the minimum number published by an author. Overall, 35 authors met this criterion. Of the 35 authors, Basset, P. is the most productive author with eleven MEMS energy harvesting papers., followed by Galayko, D., who contributed ten papers.

**Figure 4:**
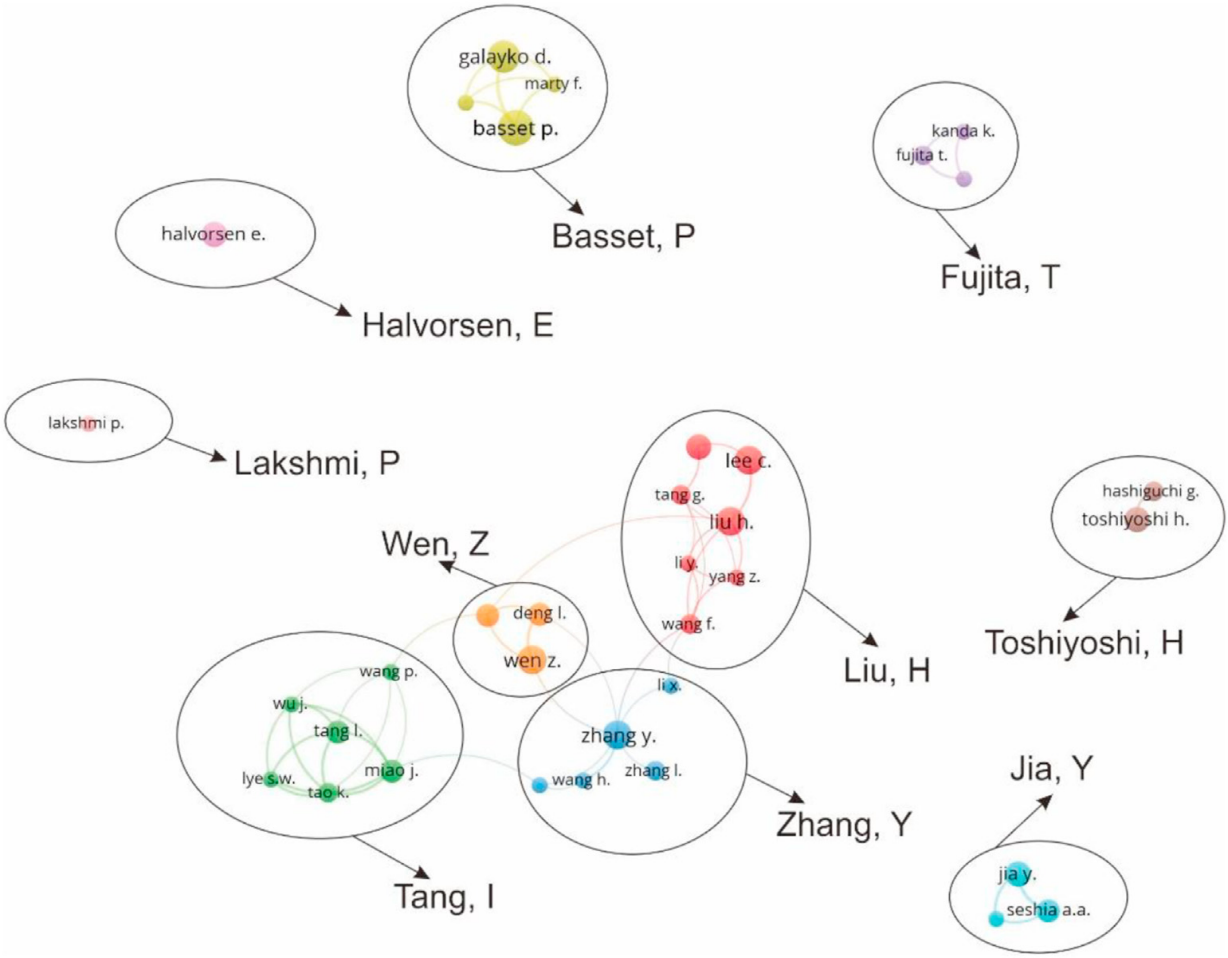
Network mapping of highly productive authors.

In general, the research group who contributed to the MEMS energy harvester can be divided into ten groups as shown in Figure 4. A productive author replaced each group's name, for example, the Zhang, Liu, Wen, and Tang groups. Figure 3 shows that the most productive research group is the Zhang, Y. group, including authors Zhang, Y., Wang, H., and Zhang, I. It is also shown the co-author network of Zhang, Y., Liu, H., Tang, I., and Wen, Z is connected. It is indicating that there is a collaboration with each other. On the other hand, the cluster of Basset, P., Toshiyoshi, H., Fujita, T., Jia, Y., Halvorsen, E., and Lakshmi, P is stand-alone, which means their cluster researched without agreement or linkage between the others.

### 3.5 Main affiliation in MEMS energy harvester research

From Table 2, we can see the top 10 institutions that have published the most MEMS energy harvester studies, including Ministry of Education China (27 papers), National University of Singapore (21 papers), University of Tokyo (18 papers), Chongqing University (17 papers), Chinese Academy of Sciences (14), Shanghai Jiao Tong University (14 papers), Nanyang Technological University (13 papers), University of Cambridge (13 papers), Sorbonne University (12 papers), and National University of Ireland (12 papers). Overall, the mainstream affiliations publishing MEMS energy harvester research are from Asia, with China lead the contribution.

**Table 2:** Top 10 Main affiliations in MEMS energy harvester research (2009–2020).

No	Author Affiliation	Number of Papers
1	Ministry of Education China	27
2	National University of Singapore	21
3	University of Tokyo	18
4	Chongqing University	17
5	Chinese Academy of Sciences	14
6	Shanghai Jiao Tong University	14
7	Nanyang Technological University	13
8	University of Cambridge	13
9	Sorbonne University	12
10	National University of Ireland	12

### 3.6 Highly cited papers in MEMS energy harvester research

Table 3 summarizes the top ten most highly cited papers in MEMS energy harvester during 11 years based on TC (total citations) and AC/Y (average annual citation per year) parameter. Figure 4 shows that cluster Zhang, Y., cluster Liu, H., and cluster Basset, P., have led a series of studies in MEMS energy harvester (5 out of 10 selected articles). In addition, the most highly AC/Y were from Zhang's paper entitled “Micro electrostatic energy harvester with both broad bandwidth and high normalized power density”. It was found in this paper that the essential concept of MEMS energy harvester should be used in several broadband bandwidths. Meanwhile, the most highly cited paper was Ferrari's paper entitled “Improved energy harvesting from wideband vibrations by nonlinear piezoelectric converters”, published in Sensors and Actuators, A: Physical in 2010, with 350 total citations and annual citations of 35. This study reported the improvement of the piezoelectric energy harvesting device.

**Table 3:** The top ten highly cited MEMS energy harvester papers from 2009 to 2020.

No	Title of the Paper	Researchers	Journals	Year	TC	AC/Y
1	Improved energy harvesting from wideband vibrations by nonlinear piezoelectric converters	Ferrari, M., Ferrari., V, Guizzetti., M, Andò., B, Baglio., S, Trigona., C	Sensors and Actuators, A: Physical	2010	350	35
2	Piezoelectric MEMS energy harvester for low-frequency vibrations with wideband operation range and steadily increased output power	Liu, H.,Tay, C.J., Quan, C., Kobayashi, T.,Lee, C.	Journal of Microelectromechanical Systems	2011	250	27.8
3	Ultra-wide bandwidth piezoelectric energy harvesting	Hajati, A., Kim, S.-G.	Applied Physics Letters	2011	197	21.9
4	Equivalent circuit modeling of piezoelectric energy harvesters	Yang, Y., Tang, L.	Journal of Intelligent Material Systems and Structures	2009	183	16.6
5	A batch-fabricated and electret-free silicon electrostatic vibration energy harvester	Basset, P., Galayko, D., Paracha, A.M., Marty, F., Dudka, A., Bourouina, T.	Journal of Micromechanics and Microengineering	2009	168	15.3
6	Investigation of a MEMS piezoelectric energy harvester system with a frequency-widened-bandwidth mechanism introduced by mechanical stoppers	Liu, H., Lee, C., Kobayashi, T.,Tay, C.J., Quan, C.	Smart Materials and Structures	2012	161	20.1
7	Design, fabrication, and characterization of CMOS MEMS-Based thermoelectric power generators	Xie, J., Lee, C., Feng, H.	Journal of Microelectromechanical Systems	2010	156	15.6
8	Micro electrostatic energy harvester with both broad bandwidth and high normalized power density	Zhang, Y., Wang, T., Luo, A., Hu, Y., Li, X., Wang, F.	Applied Energy	2018	140	70
9	Acoustic energy harvesting using resonant cavity of a sonic crystal	Wu, L.-Y., Chen, L.-W., Liu, C.-M.	Applied Physics Letters	2009	133	12.1
10	A micro electromagnetic low level vibration energy harvester based on MEMS technology	Wang, P., Tanaka, K., Sugiyama, S.,Dai, X., Zhao, X., Liu, J	Microsystem Technologies	2009	131	11.9

### 3.7 Keywords analysis

**Figure 5:**
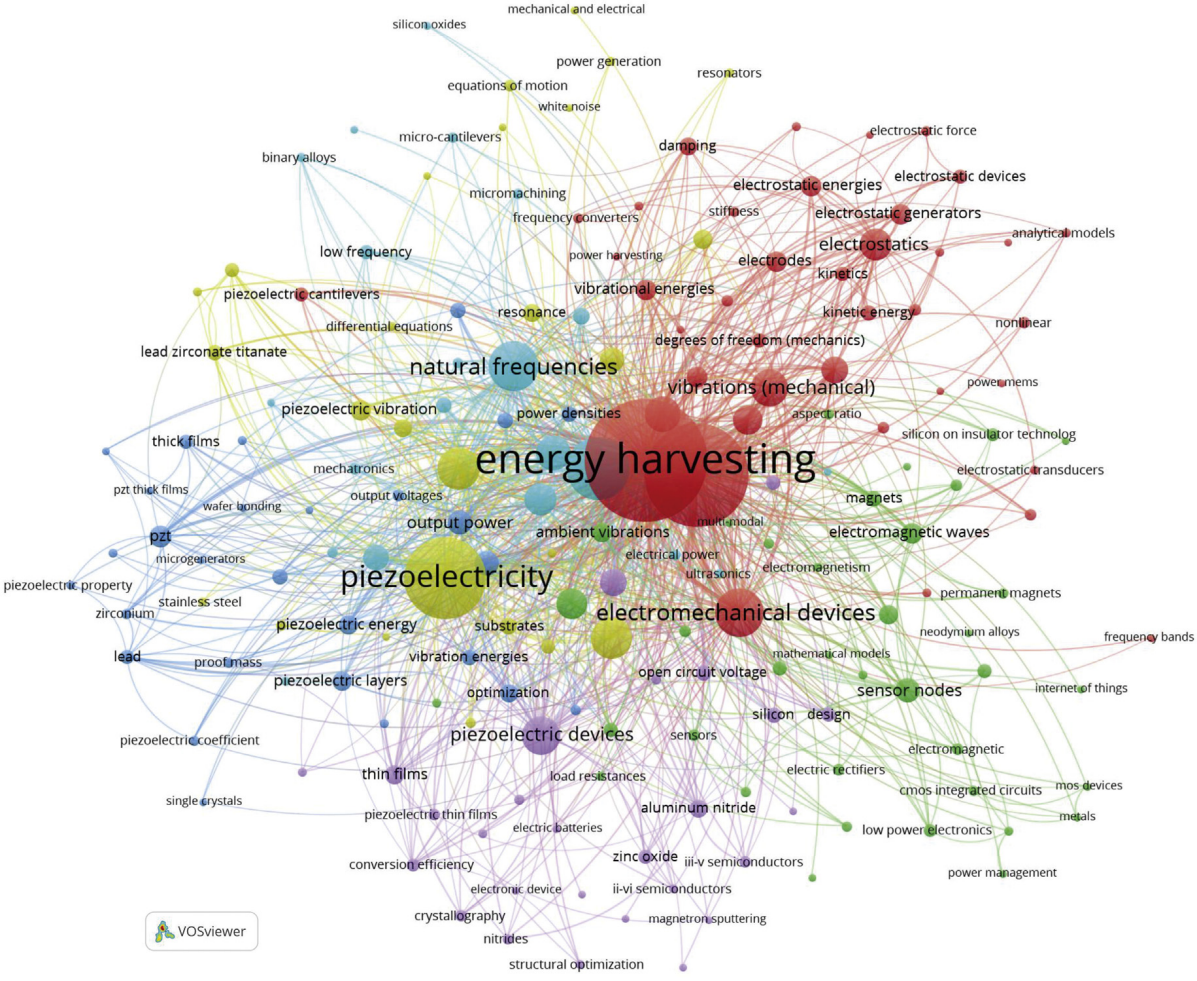
High-frequency keywords network visualization map.

To highlight the paper's central focus and make it easier for the readers to map, the keywords are essential needed (Jin et al., 2019). The sample keywords are explored and analyzed using VOSviewer software to show the high frequency of keywords and their relationships. The minimum number of keywords was set to 5 and 25 of the keywords appear in Figure 5.

The word “Energy Harvesting” was the most mentioned keyword in conjunction with “electrostatics” “damping”. Besides, “piezoelectricity, piezoelectric devices, natural frequencies, sensor nodes” were also frequently mentioned in the MEMS energy harvester literature. The results also show certain key topics attracting huge attention related to different dimensions, such as materials and actuator concepts (i,e., piezoelectric, electrostatic, electromagnetic), method (i.e., finite element analysis, nonlinear). The interrelationship among those keywords is also helpful for understanding MEMS energy harvester subjects over the last 11 years.

**Figure 6:**
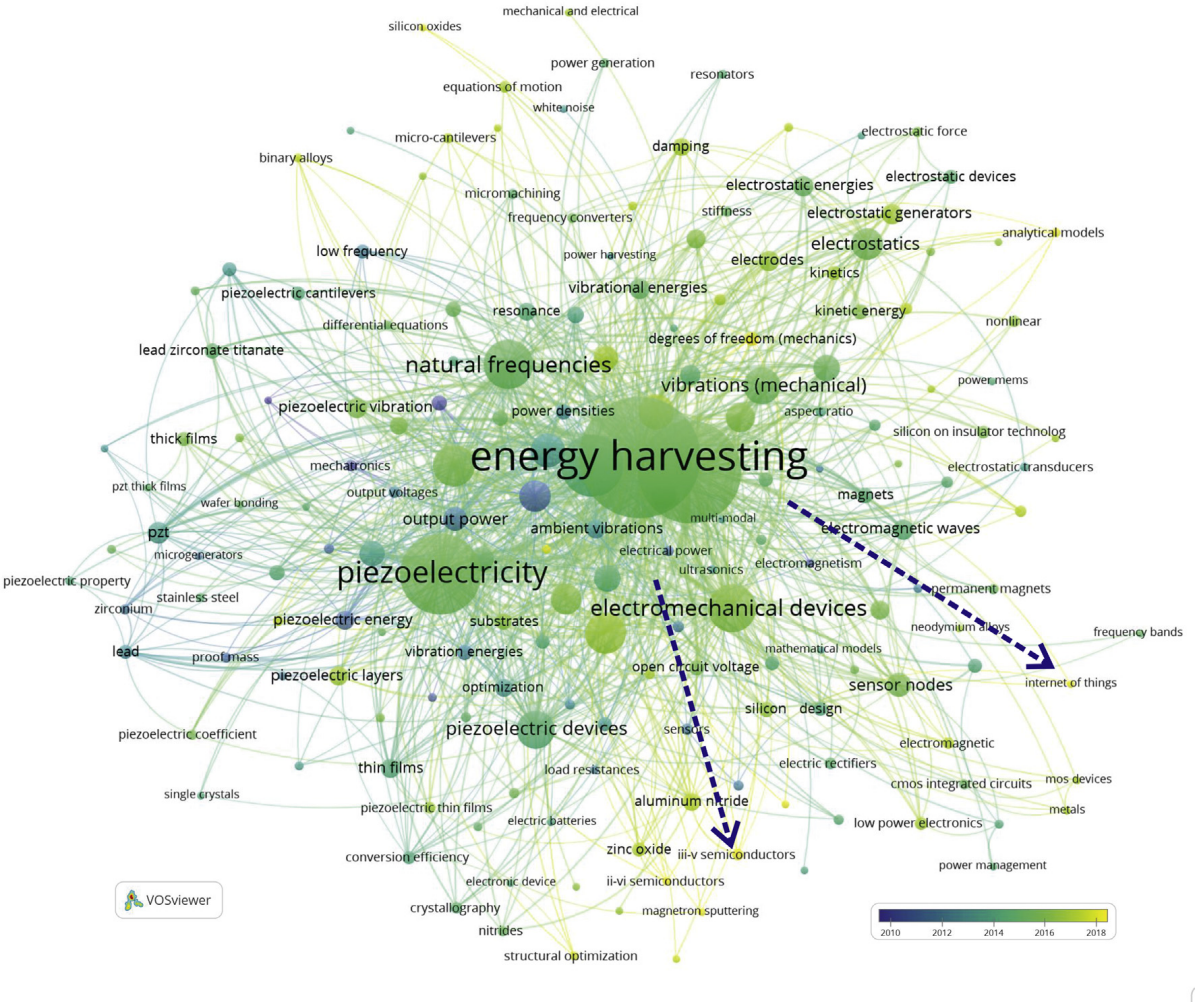
High-frequency keywords overlay visualization map.

It is worth understanding the evolutionary process of high-frequency keywords in MEMS energy harvester research in the past decade. Thus, the researchers will be more comfortable identifying the main research trends. Figure 6 shows the keywords trends in MEMS energy harvester research in the past decade. The circle's colour indicates the occurred time slot clarity, with darker circlers exhibits earlier highlighted keywords. The two blue dot arrows show the high-frequency keywords shift in the last 11 years. For instance, the colour of the piezoelectricity circle is light green. Following the colour references in the bottom right corner of the figure, we can see that those highlighted keywords happened in about 2014. In general, it is indicated that the researcher pays more attention to these topics at that time. Furthermore, the keywords that may appear in the future related to MEMS energy harvester topics are the internet of things and III-V semiconductors.

### 3.8 Future research in MEMS energy harvesting

At present, most of the MEMS energy harvester is based on piezoelectric materials (Tian et al., 2018) such as single piezoelectric crystals (Li et al., 2014) and piezoelectric ceramics (polycrystalline), and polyvinylidene fluoride (PVDF) (Song et al., 2017). Moreover, through special processing, including nanotubes, nanowires, nanoparticles, and materials such as magnetics and ceramics (Shi et al., 2017), some organic nanostructures are also found to contribute to the development of MEMS energy devices.

Analysis on VOSViewer confirmed these statements and based on the several mapping analysis above; there are several potential directions in future MEMS energy harvester research identified. The typical MEMS energy harvester is based on piezoelectric, electrostatic and electromagnetic energy conversion mechanisms, while and the ceramics piezoelectric material contributes to the rapid development of the energy harvester device. Therefore, the other piezoelectric materials energy harvester's variation and investigations are interesting to investigate deeply more. The cantilever beam is a common structure for maximizing the MEMS energy harvesters output power. Meanwhile, the resonant frequency needs to be adjusted to match the excitation energy. Therefore, the shape and structure of the cantilever beam and the method to broaden the frequency bandwidth of the MEMS energy harvester need more study. The other MEMS energy harvester method, such as electromagnetic and electrostatic, can be the alternative principle since the piezoelectric materials are more investigated simultaneously. Finally, the vibrational energy capture implementing magnetic, ceramics, and polymers have been revealed as the potential method and materials playing an essential role for the next energy conversion.

## 4 Conclusions

In this paper, an analysis of MEMS energy harvester research's impact over the past decade aims to provide a guideline for future MEMS research in the energy field. VOSviewer software has been used to visualize and analyze the bibliometric networks compiled from the initial source data that appeared in various Scopus publications. Based on the analysis on 672 papers MEMS energy harvester research published from 2009 to 2020, the bibliometric analysis showed that the journals publishing research reports on MEMS energy harvester mainly cover the engineering field, materials and physics science and mostly published in high impact journal, such as JMEMS and Journal of Micromech. and Microeng. The authors and affiliations from China, the USA and Europe are the main contributors to MEMS energy harvester research in which the highest total citation of 350 since 2010 was revealed. An analysis of the research topic interprets that the keywords such as MEMS, micromachines, microsystem, energy harvester, power harvesting and energy scavenging have long been focused on by MEMS for energy harvester. The evolution, trends and future direction based on review and the overlay visualization map of keywords give a useful guide to future MEMS energy harvester research.

## Declarations

### Author contribution statement

All authors listed have significantly contributed to the development and the writing of this article.

### Funding statement

10.13039/501100009509Kementerian Riset dan Teknologi/Badan Riset dan Inovasi Nasional Republik Indonesia through the Penelitian Terapan Grant 2020-2022 (473/UN40.D/PT/2020).

### Data availability statement

Data included in article/supp. material/referenced in article.

### Declaration of interests statement

The authors declare no conflict of interest.

### Additional information

No additional information is available for this paper.
